# Effective healthcare teams require effective team members: defining teamwork competencies

**DOI:** 10.1186/1472-6963-7-17

**Published:** 2007-02-07

**Authors:** Sandra G Leggat

**Affiliations:** 1 School of Public Health, La Trobe University, Victoria, 3086, Australia

## Abstract

**Background:**

Although effective teamwork has been consistently identified as a requirement for enhanced clinical outcomes in the provision of healthcare, there is limited knowledge of what makes health professionals effective team members, and even less information on how to develop skills for teamwork. This study identified critical teamwork competencies for health service managers.

**Methods:**

Members of a state branch of the professional association of Australian health service managers participated in a teamwork survey.

**Results:**

The 37% response rate enabled identification of a management teamwork competency set comprising leadership, knowledge of organizational goals and strategies and organizational commitment, respect for others, commitment to working collaboratively and to achieving a quality outcome.

**Conclusion:**

Although not part of the research question the data suggested that the competencies for effective teamwork are perceived to be different for management and clinical teams, and there are differences in the perceptions of effective teamwork competencies between male and female health service managers. This study adds to the growing evidence that the focus on individual skill development and individual accountability and achievement that results from existing models of health professional training, and which is continually reinforced by human resource management practices within healthcare systems, is not consistent with the competencies required for effective teamwork.

## Background

Teamwork is essential in the provision of healthcare. The division of labor among medical, nursing and allied health practitioners means that no single professional can deliver a complete episode of healthcare [1]. Yet there is little formal training in teamwork skill development in undergraduate or postgraduate health professional education programs – teamwork skills are largely learned 'on-the-job' [2]. In healthcare, where patient outcomes are dependent on effective interdisciplinary teamwork, there is need for better preparation of health professionals in teamwork.

Although many studies have identified teamwork as a requirement for high quality, safe patient care [3-7], within healthcare we have limited understanding of how individual health professionals contribute to effective teamwork. While there has been substantial study attempting to identify and define the requirements for effective healthcare teams, the predominant focus has been on improving existing teams [8-10]. There has been little research into the educational and training needs of healthcare professionals to enhance their participation in workplace teams; healthcare team members do not understand the personal competencies required for team success [11]. To assist in planning formal education programs this study aimed to identify the competencies held by healthcare professionals that were perceived by health service management colleagues to enhance teamwork. It has been suggested that "each team member's abilities, skills experience, attitudes, values, role perceptions and personality – all the things that make a person unique – determine what they are willing and able to contribute, their level of motivation, methods of interaction with other group members and degree of acceptance of group norms and the organization's goals" [[12], p. 676]. This suggests the need to focus on individual characteristics that have been found to contribute to teamwork, as "pre-requisite characteristics of effective teamwork" [[13], p. 204].

Generally understood as the clusters of skills, abilities and knowledge needed for occupational tasks competency-based health professional education has had a long history and is stressed in the accreditation of healthcare management education programs world wide [14-17]. In addition, there is increasing evidence that management competencies are an important source of competitive advantage for organizations [18,19]. Given the long standing focus on competency requirements for health service managers this study aimed to identify the competencies that were seen by health service managers to be related to effective teamwork within a health service workplace.

## Methods

### Study framework

A systematic literature review was conducted using standard literature search techniques for the years 1995 to 2005. Online computer searches of relevant computerised bibliographic databases were completed, using the key words "team, teamwork, inter-professional collaboration, multidisciplinary, competency and communities of practice". These computer searches were supplemented by exploration of documented teamwork competencies used for human resources management and management education purposes.

The research tends to be focused on improving team performance but few studies were identified with either randomised or control methods that enabled generalisation. In addition studies tended to rely on subjective measures of team performance [20]. This is confounded by the finding that team members tend to be overly positive in their assessment of the performance of their group; yet this positive performance assessment is not generally supported by objective performance measures [21]. There were no sound empirical studies that confirmed the teamwork competencies related to successful teamwork performance, in general, or specifically related to health care. Therefore to develop the competency framework to be tested in this study a large number of experimental and non-experimental papers were reviewed with the intent of building a model that was comprehensive in outlining potential teamwork competencies as it was not possible to develop a model that was empirically supported.

**Figure 1:**
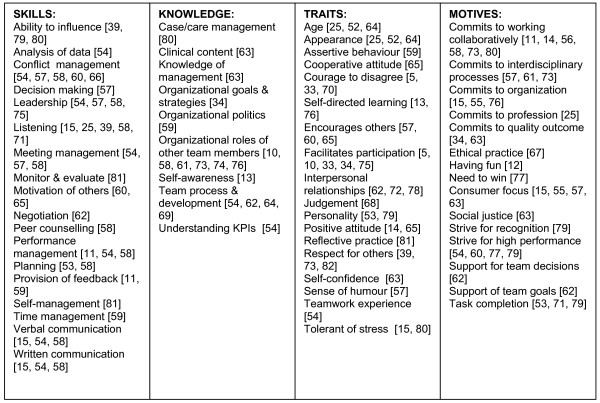
**Possible skills, knowledge, traits and motives influencing teamwork**. A figure illustrating the findings of the literature review used to develop the potential competencies explored in this paper. The reference list for the competencies in Figure 1 appear in the main reference list [5, 10-15, 25, 33, 34, 39, 52-82] (i.e. [5] [10] [11] [12] [13] [14] [15] [25] [33] [34] [39] [52] [53] [54] [55] [56] [57] [58] [59] [60] [61] [62] [63] [64] [65] [66] [67] [68] [69] [70] [71] [72] [73] [74] [75] [76] [77] [78] [79] [80] [81] [82])

Management competencies, the basis for healthcare management education, are generally considered to comprise skills, knowledge, traits (including attitudes) and motives (including values)[22,23], and therefore these four categories formed the basis for the model. Through the detailed literature review 18 skills, nine knowledge areas, 18 traits and 15 motives were identified as having relevance for effective teamwork (see Figure 1).

### Survey

Survey methodology was used to ascertain the competencies perceived by health service managers to contribute to effective team participation in healthcare. The survey respondents were requested to identify the top quartile of each of the skills (5 out of 18), knowledge (3 out of 9), traits (5 out of 18) and motives (4 out of 15) from the lists in Figure 1 that they perceived to be most associated with the effective performance of a member of a management/administrative or clinical care team in which they participated. The ranking approach was used as it has been shown to result in higher quality data than ratings [24]. Instead of ranking the entire list, the respondents were requested to identify only the top quartile to limit the primacy effect often found with ranking studies [24]. The reliability analysis provided Cronbach alpha of over 0.8.

Based on the nature of health care, where clinical care teams are the main work teams responsible for producing healthcare services, and management teams are responsible for the organizational work through coordination and direction setting [25], the respondents were asked to focus their responses to either a management or a clinical team in which they had participated. The respondents were asked to only consider ongoing teams; project and other teams with one-time outputs were not included. Ethics approval was granted from the La Trobe University Ethics Committee. Data analysis comprised descriptive statistics and univariate chi-square using SPSS.

### Study population

Pre-testing was completed by a small sample of five individuals drawn from the target population to identify the time requirements to complete the questionnaire and ensure the clarity of the questions. The questionnaire was then distributed by email to the 680 members of the Victorian State Branch of the Australian College of Health Service Executives (College). Membership of this College requires current study in or completion of an accredited health service management course and/or employment as a senior health service manager in a recognized position. Based on error messages in transmission and emails returned to source it was estimated that around 600 members received the emailed questionnaire. The completed surveys were emailed to an administrative mailbox where all identifying information was removed before the surveys were provided for data entry and analysis. Eleven completed questionnaires were returned by post.

## Results

Following emailed reminders, 224 completed surveys were returned for a 37% response rate. The demographics of the respondents (Table 1) were representative of the sampled population, with 132 (60%) female and 90 (40%) male. Two surveys were returned without the demographic information completed. The majority of the respondents were senior managers (n = 114 51.4%), with 52 Chief Executive Officers (CEOs) (23.4%), 33 middle managers (14.9%) and 23 (10.4%) describing themselves as 'other'. The majority (60.8%) of the respondents reported an age range of 40 to 59 years.

**Table 1:** Reported age range, gender and position

Position	CEO		Senior Manager		Middle Manager		Other		Total	
Age/Sex	F	M	F	M	F	M	F	M	#	%

20–39 yrs	4	9	28	14	7	3	8	4	77	34.7
40–59 yrs	14	25	46	16	14	9	5	6	135	60.8
60+ yrs	0	0	6	4	0	0	0	0	10	4.5
Total	18	34	80	34	21	12	13	10	222	100

As expected from this sample of members of a professional management organization, the majority (n = 198 88%) choose a management team as the focus for the questionnaire. It was surprising that any of the respondents choose a clinical team, but there were 26 (12%) completed questionnaires that focused on a clinical care team. Only four males completed their questionnaire based on a clinical team, perhaps indicative of the greater proportion of male respondents in CEO and senior management positions. The sample size of 26 is small for the clinical team respondents and therefore the clinical team results cannot be considered to have the same validity as the management team results and are not reported. However the fact that the respondents identified different competencies for the clinical and management teams lends some support for previous study that has shown that different team types will have different determinants of effectiveness [25].

The respondents were asked to consider team success as achievement of team goals and team member satisfaction. The respondents were requested to identify from the list in Figure 1 the skills, knowledge, traits and motives that the most effective team members demonstrated and that they had observed to enhance team performance. Respondents were also given the opportunity to add to the lists. There were three additions: project management was cited by one respondent as an important skill, and tenacity and tolerance of ambiguity were added as relevant traits.

While the primary purpose of this study was to identify a set of competencies, post hoc analysis of the data suggested differences in responses on two variables of gender and position within the organization, confirmed by chi square analysis. These post hoc analyses were chosen as previous study has found differences between the approaches of male and female managers [26,27] and the requirement for different competencies among different levels of health service managers [17,28,29].

The purpose of this study was to identify the competencies that were seen by health service managers to be related to effective teamwork within a health service workplace. To identify the competencies the data analysis focused on the characteristics that were identified by more than 50% of the respondents. This approach is consistent with previous teamwork study by McDonough who found that in each category one variable was mentioned much more frequently than the others by the study respondents [30]. Those competencies that were identified by more than 50% of the respondents were likely to represent the competencies thought most important by the respondent sample.

### Skills

The ability to perform an activity, a skill, can be the result of natural talent or acquired through education or training. As shown in Table 2 only one skill, *leadership*, was identified as important by more than 50% of the respondents. The differences in responses by sex (n = 198) were examined and only two skill areas were found to be related to the sex of the respondent. The male respondents were significantly more likely than the female respondents to identify *ability to influence *as an important skill (χ^2 ^= 7.490 1 df p = 0.006), while the female respondents were significantly more likely to identify *negotiation *as an important skill (χ^2 ^= 5.878 1 df p = 0.015). However when the analysis was completed by position the female CEO respondents were significantly less likely than the female respondents in other positions to identify *negotiation *as an important skill (χ^2 ^= 8.006 3 df p = 0.046).

**Table 2:** Skill frequencies

SKILLS	#	%
Ability to influence	89	44.9
Analysis of data	90	45.5
Conflict management	17	8.6
Decision making	82	41.4
Leadership	116	58.6
Listening	82	41.4
Meeting management	25	12.6
Monitoring & evaluation	37	18.7
Motivation of others	55	27.8
Negotiation	48	24.2
Peer counseling	12	6.1
Performance mgmt	15	7.6
Planning	76	38.4
Provision of feedback	37	18.7
Self-management	56	28.3
Time management	20	10.1
Verbal communication	76	38.4
Written communication	26	13.1

The percentages are based on the number of respondents.

### Knowledge

As shown in Table 3 the knowledge area with over 50% response was knowledge of *organizational goals and strategies*. Female respondents were significantly more likely than male respondents to identify *self-awareness of strengths and weaknesses *as important knowledge (χ^2 ^= 15.172 1 df p = 0.000).

**Table 3:** Knowledge frequencies

KNOWLEDGE	#	%
Case/care management	31	16
Clinical knowledge	52	26.8
Management knowledge	59	30.4
Org goals & strategies	114	58.8
Organizational politics	71	36.6
Roles of team members	41	21.2
Self-awareness	83	42.8
Team development	63	32.5
Understanding of KPIs	60	30.9

The percentages are based on the number of respondents.

### Traits

Individual characteristics or traits may be highly visible demographic characteristics such as age or appearance, or may be less apparent, such as attitudes. In regards to traits the management team respondents identified *respect for others *as the most important trait (Table 4).

**Table 4:** Trait frequencies

TRAITS	#	%
Age	6	3
Appearance	2	1
Assertive behaviour	28	14.1
Cooperative attitude	94	47.5
Courage to disagree	90	45.5
Self-directed learning	32	16.2
Encourages others	53	26.8
Facilitates participation	85	42.9
Interpersonal relations	54	27.3
Judgment	61	30.8
Personality	27	13.6
Positive attitude	92	46.5
Reflective practice	38	19.2
Respect for others	112	56.6
Self-confidence	39	19.7
Sense of humour	81	40.9
Teamwork experience	41	20.7
Tolerant of stress	33	16.7

The percentages are based on the number of respondents.

The female respondents were significantly more likely than the male respondents to consider *positive attitude *as an important trait (χ^2 ^= 7.154 1 df p = 0.007). The male senior and middle management respondents were significantly more likely to include *self-directed learning *(χ^2 ^= 22.721 3 df p = 0.000) than the CEO and other male respondents. The male CEO and female senior manager respondents were less likely to include *respect for others *(male: χ^2 ^= 13.810 3 df p = 0.003, female: χ^2 ^= 11.592 3 df p = 0.009) as a trait as compared to the respondents from the other male and female management categories.

### Motives

This group included perceived intrinsic values and personal motives. The most important motives seen to contribute to the success of the team were *commitment to working collaboratively*, *commitment to the organization *and *commitment to a quality outcome *(Table 5).

**Table 5:** Motive frequencies

MOTIVES	#	%
Work collaboratively	127	64.1
Interdisciplinary	28	14.1
Commit to organization	128	64.6
Commitment to profession	35	17.7
Quality outcome	137	69.2
Ethical practice	57	28.8
Having fun	39	19.7
Need to win	10	5.1
Consumer focus	98	49.5
Social justice	38	19.2
Strive for recognition	8	4.0
High performance	83	41.9
Support team decisions	62	31.3
Support of team goals	49	24.7
Task completion	73	36.9

The percentages are based on the number of respondents.

There were no significant motive differences among male and female respondents. The CEO respondents for both males and females were significantly more likely to indicate *commitment to organization *(male: χ^2 ^= 13.553 3 df p = 0.004 female: χ^2 ^= 13.031 3 df p = 0.005) and significantly less likely to indicate *task completion *(male: χ^2 ^= 20.426 3 df p = 0.000 female: χ^2 ^= 11.106 3 df p = 0.011) as key motives.

## Discussion

The respondent sample reflected the population of health service managers in the State of Victoria, Australia. The survey respondents were requested to identify the competencies that they had seen in other team members which enhanced teamwork. We know that individuals often identify characteristics in others that are most like their own characteristics [31] which suggests that there is a chance the respondents reported characteristics most like the ones they themselves possessed and that were not necessarily related to effective teamwork. This risk was minimized by the questionnaire design that asked respondents to focus on an identified team, and an individual other then themselves who they perceived as having a positive impact on the team, and reporting on what they had observed. Another limitation is that the survey forced the respondents to identify their priorities within each of the skills, knowledge, traits and motives categories; while a set of competencies from all of these areas was identified, further study is required to rank the importance among the identified skills, knowledge, traits and motives.

### Management and clinical teams may require different competencies

While the under-representation of clinical care teams in the sample made it difficult to test if the observed differences in the reported competencies between the management and clinical teams were statistically significant, the fact that a sample of health service managers distinguished between competencies for clinical and management teams is an important finding and needs further study. Previous teamwork research has confirmed that the type of team influences the factors related to effectiveness [25,32] and this study suggested that within healthcare settings there are perceived differences in the competencies important for management and clinical teams.

### Management team competencies

The management team competencies were strongly consistent with previous studies on teamwork performance. Three motives received the highest ranking of all the skills, knowledge, traits and motives; *commitment to working collaboratively *(64.1%), *commitment to a quality outcome *(69.2%) and *commitment to organization *(64.6%). Although not unequivocally supported through controlled experimental design, cross-sectional and case studies have suggested that teams with a climate of psychological safety [5,33] that encourages high levels of participation [34] toward clear goals [35,36] that enable high performance and quality expectations [34,37,38] demonstrate better team performance [33,39]. The respondents in this study consistently identified three motives that reflected these previous findings.

This study approached the issue of team performance from a different perspective than previous studies. Instead of team level analysis we focused on the perceptions of health care managers of individual characteristics that contributed most to team success, and yet the results still supported previous study. From the perspective of these respondent managers, individuals participating in management teams in health care organizations were considered to have the greatest impact on team performance when they demonstrated commitment to working collaboratively, commitment to the organization and commitment to a quality outcome. Of all of the skills, knowledge, traits and motives that were provided, the fact that over 60% of the management team respondents indicated the importance of these three motives lends strong support for team members who:

• demonstrate their commitment to the organization by communicating organizational goals and objectives and assisting their team colleagues to translate the needs of the organization into performance outcomes for the team, and

• demonstrate their commitment to collaboration and quality by facilitating the needed psychological safety among team members that enables them to discuss and learn from mistakes and to challenge their team colleagues when it is required for a quality outcome.

It is clear that more methodologically and statistically rigorous investigation is required to better confirm the relationships between the competencies identified in this study and the performance of teams in health sector organizations. While progressive human resource management promotes participation, training and teamwork consistent with the identified management competencies, healthcare organizations do not always provide best practice HRM [40-42], and the people side of management has often been ignored in the pursuit of health reform [43,44]. In addition the traditional training and socialisation of health professionals tends to emphasise individual skills, accountability and achievement [45] and the healthcare system continues to foster individual [46] and discipline-specific rewards, supervision and education which consistently leads to difficulties with collaboration across professions, and reliance on hierarchy to manage coordination needs and mediate conflict [42]. The strong support for leadership skills among management team members in this study holds up the notion that within healthcare, leadership that rests at the top of an authority hierarchy needs to be refocused to develop leaders throughout the organization [47]. There needs to be a radical shift in HRM practice [40] in health care to train, performance manage and reward practices that result in clinical and management leaders through the organization – leaders that can foster the organizational commitment and psychological safety that is likely to improve teamwork outcomes.

In healthcare, employee relationships and behaviours are often influenced by the highly professional nature of the workforce, where there is often stronger alliance to the profession than to the organization. Many managers are professionally trained clinicians [48], and they often continue clinical practice even when they have assumed a management role. The findings of this study reinforce previous research that has identified the need for management training of clinician leaders [49-51]. Although many of the competencies developed in clinical education and ongoing clinical practice are transferable to management, there are skill and knowledge deficits [51]. The transition from clinician to manager requires a substantial cognitive shift from a primary commitment to individual care to a community/organizational focus [28].

This study also highlighted differences in perceptions among male and female health service managers that may influence team behaviours and ultimately team effectiveness. The differences noted in the responses of the male and female managers appear consistent with previous study.

For example, male leaders have been found to be more transactional and derive their power from their position on the formal organizational structure [26]. In contrast, women tend to be more transformational and derive their power from personal characteristics. In this study the male respondents demonstrated this transactional nature, identifying ability to influence as a key teamwork skill, while the female respondents suggested negotiation, self-awareness of strengths and weaknesses and positive attitude were important. Studies have suggested that women managers in male-dominated industries employ more 'masculine' management characteristics [27], and we found similarities between the women in CEO positions and male respondents. For example, the female CEOs were less likely than the female respondents at other organizational levels to identify negotiation as an important teamwork skill. While more study of these differences and the impact on team performance is warranted, these results provide some support for the differences in styles of male and female managers.

Different levels of health service managers require different competencies. It has been suggested that front line and/or entry level positions rely on technical expertise, middle managers require greater skills in human resource management and the senior level roles need greater conceptual skills [17,28]. Often the senior roles are thought to focus more on managing output related organizational adaptation and change [29], while junior levels manage the technical operational aspects of the organization. The findings provided some support. The CEO respondents were more likely to stress commitment to the organization and less likely to indicate task completion. The senior and middle managers focused more on transactional skills, such as negotiation and these manager respondents also stressed self-directed learning to a greater extent.

## Conclusion

This study has explored the individual teamwork competencies perceived by health service managers to contribute to effective teamwork in management teams. The findings are consistent with other studies and support the need for a greater focus on progressive human resource management within the healthcare sector, with a focused teamwork development approach. The management team competencies suggested team success when the members displayed a strong focus on the organization and the values, climate and culture underlying effective interpersonal and teamwork relationships. The findings of this study will be used to develop focused teamwork training initiatives for health service managers.

## Competing interests

The author(s) declare that they have no competing interests.

## Pre-publication history

The pre-publication history for this paper can be accessed here:


